# Outer Membrane Vesicles Attenuate *Klebsiella pneumoniae* Infection Injury by Affecting Macrophage Polarisation and Helper T Cell Differentiation

**DOI:** 10.3390/microorganisms13122849

**Published:** 2025-12-15

**Authors:** Wei Fan, Wei Wang, Lin Kong, Shifan Chen, Xinyu Zhang, Yilu Zhai, Bo Zhang, Yan Wang, Dazhuo Zhao, Xiukai Tang, Jiaqi Fu, Fuliang Sun

**Affiliations:** 1 Department of Veterinary Medicine, College of Agricultural, Yanbian University, Yanji 133002, China; fw011118@126.com (W.F.); 2023050989@ybu.edu.cn (W.W.); konglin@ybu.edu.cn (L.K.); 18341990551@163.com (S.C.); 15043444752@163.com (Y.Z.); zb09272020@163.com (B.Z.); 13804207753@163.com (Y.W.); xtovo9@163.com (X.T.); 2022010659@ybu.edu.cn (J.F.); 2 Institute of Special Economic Animal and Plant Sciences, Chinese Academy of Agricultural Sciences, Changchun 130112, China; xinyuzhang19981124@163.com; 3 Department of Animal Disease Prevention and Control Centre, Yanji 133002, China; zhaodazhuo1980@126.com

**Keywords:** outer membrane vesicles, *Klebsiella pneumoniae*, macrophage and helper T cell polarization, adaptive immunity, immunoregulation

## Abstract

*Klebsiella pneumoniae* (*K. pneumoniae*) is an opportunistic bacteria that can result in severe liver abscesses, pulmonary damage, and potentially fatal outcomes. Research has demonstrated that the outer membrane vesicles (OMVs) released by it can provide significant protection to infected animals and may serve as a promising candidate antigen for the development of a novel vaccine. Nevertheless, the specific mechanisms through which OMVs mitigate the detrimental effects of *K. pneumoniae* infection by promoting the polarization pathways of macrophages and T helper cells (Th cells) remain poorly understood. In this study, we first confirmed that *Klebsiella pneumoniae* outer membrane vesicles (*K. pneumoniae*_OMVs) were protective in *K. pneumoniae*-infected mice, and then we investigated the protective mechanisms by transcriptome data analysis. Then, we constructed a model of in vitro macrophage polarization, an in vivo model for Th differentiation, and a *K. pneumoniae* infection model in *K. pneumoniae*_OMVs-immunized mice. qRT-PCR, IHC, Western blotting, and ELISA were used to confirm the polarization indicators. The results showed that *K. pneumoniae*_OMVs were able to provide specific protection for mice with a maximum protection rate of 80%. In addition, the results of a transcriptome analysis suggested that the protective mechanism might be related to Th cells and macrophage polarization. Mice immunized with *K. pneumoniae*_OMVs were able to achieve rapid bacterial clearance after *K. pneumoniae* infection through an M1/Th1 immune response. Subsequently, tissue repair was accomplished through Th2/M2 immune response in the late stage of *K. pneumoniae* infection to avoid causing inflammatory damage. This study offers a theoretical foundation for the *K. pneumoniae*_OMVs vaccine’s actual application.

## 1. Introduction

One of the six superbugs (ESKAPE) is *K. pneumoniae*, a common opportunistic infection. This condition predominantly affects individuals with compromised immune systems and those presenting with preexisting medical disorders. The primary manifestations of infection include urinary tract infections, bloodstream infections, and respiratory tract infections. Such infections are generally linked to elevated rates of morbidity and mortality [1,2]. Antibiotics are routinely used to deal with *K. pneumoniae* infection, but unregulated clinical use has resulted in the proliferation of drug-resistant *K. pneumoniae*. The World Health Organization has identified this condition as one of the dangers to public health since it makes treatment more challenging [3]. Finding alternate treatments or preventative measures is therefore imperative. Gram-negative bacteria spontaneously release physiologically active vesicle-like molecules called OMVs [4]. Recent research demonstrates that, relative to traditional vaccines, the diverse pathogen-associated molecular patterns present within OMVs elicit multifaceted immune responses, thereby conferring targeted protection against infection in murine models [5,6]. This effect has been observed with OMVs derived from *Neisseria meningitidis* [7], *Escherichia coli* [8], and *Helicobacter pylori* [9]. However, the exact mechanisms by which these OMVs mediate immune protection have yet to be fully elucidated.

Macrophages play a critical role in the innate immune system’s defense against pathogenic pathogens [10]. Under different environmental stimuli, macrophages can be polarized into two different phenotypes: classically activated macrophages (M1) and alternatively activated macrophages (M2) [11]. Among these, M2 macrophages are connected to the organism’s reparative and anti-inflammatory processes, whereas M1 macrophages are linked to pro-inflammatory reactions [12]. Resistance to bacterial infection is significantly influenced by macrophages’ M1-like polarization [13]. In the context of treating bacterial pneumonia, studies have shown that carbon dots can promote macrophage polarization toward the M1 phenotype via the PI3K/AKT/mTOR signaling pathway [14]. Macrophage M2 polarization facilitates the intracellular replication of Salmonella and contributes to systemic infection; however, it also plays a positive role in anti-inflammatory responses and tissue repair during the later stages of infection [15]. Numerous cytokines secreted by macrophages in various polarization states encourage naive CD4+ T cells to differentiate into Th1 and Th2 subsets [16]. IL-12, secreted by M1 macrophages, promotes the activation of Th1 cells by binding to specific receptors on the surface of Th cells. For intracellular infections to be eliminated, Th1 cells are essential. At the same time, anti-inflammatory substances generated by M2 macrophages stimulate humoral immunity, boost B cells’ ability to make antibodies, and encourage Th cells to differentiate into Th2 cells. However, these factors also suppress Th1 cell-mediated inflammatory responses [17,18]. As a result, the M1/M2 and Th1/Th2 subpopulations may operate synergistically to address pathogen infections.

Research indicates that OMVs coated with nanoglycyrrhizic acid can induce mixed Th1/M1 and Th2 immune responses, thereby protecting infected mouse models [19]. In contrast, the OMVs secreted by *Pseudomonas aeruginosa* exert a protective effect by promoting Th2-dominant humoral immune responses while simultaneously suppressing the generation of excessive inflammatory responses [20]. Similarly, *K. pneumoniae*_OMVs can prevent mortality in *K. pneumoniae*-infected mice by inducing humoral and cellular immune responses [21]. However, the specific mechanisms underlying their immunoprotective effects, particularly about the modulation of Th cell and macrophage polarization, remain inadequately understood. Consequently, this research seeks to examine the impact of *K. pneumoniae*_OMVs on the polarization of macrophages and the differentiation of Th cells, in addition to elucidating the immune protective mechanisms in mice infected with *K. pneumoniae* via these pathways. The purpose of this study is to provide a theoretical framework for the practical application of *K. pneumoniae*_OMVs vaccines.

## 2. Materials and Methods

### 2.1. Bacterial and Cell Culture

Clinical isolates of *K. pneumoniae* (FW-1) and *Proteus mirabilis* (*P. mirabilis*) (*PM-1*) strains were cryopreserved at the College of Agriculture, Yanbian University (Yanji, China). The 16S rRNA gene sequences of the strains utilized in this study (accession numbers: PV248949, PQ810001) have been deposited in the GenBank database. *K. pneumoniae* was cultivated in Luria–Bertani medium at a consistent temperature of 37 °C to achieve the necessary concentration for the extraction of *K. pneumoniae*_OMVs and for conducting the mouse infection assay. The Raw264.7 cell line was obtained from the Pathology Laboratory of the College of Agriculture at Yanbian University. The Raw264.7 cells were maintained in Dulbecco’s Modified Eagle Medium (DMEM) supplemented with 10% fetal bovine serum (FBS) and antibiotics, specifically penicillin (100 U/mL) and streptomycin (100 U/mL), throughout the duration of the experiment.

### 2.2. Extraction of OMVs

**Figure 1:**
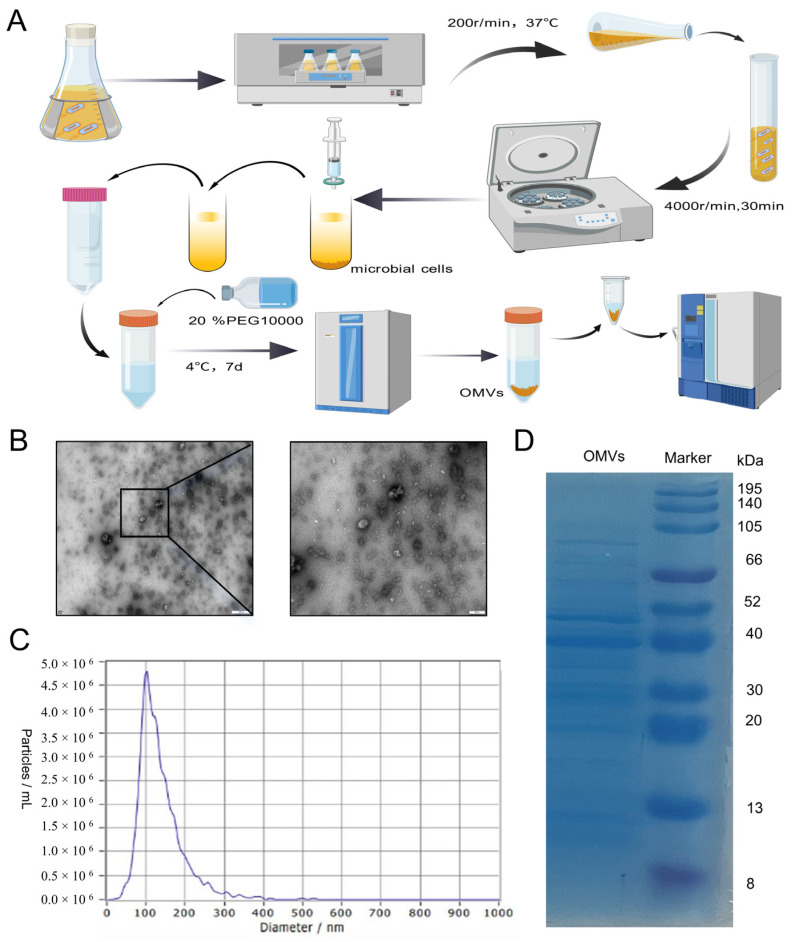
The characterization of *K. pneumoniae*_OMVs. (**A**) A schematic representation of the extraction process for *K. pneumoniae*_OMVs, created using BioGDP.com [22]. (**B**) Transmission electron microscopy images of *K. pneumoniae*_OMVs, observed at a magnification of 100 and 500 nm. (**C**) The particle size distribution of *K. pneumoniae*_OMVs. (**D**) The results of protein blotting of *K. pneumoniae*_OMVs.

The procedure for the extraction of *K. pneumoniae*_OMVs is illustrated in Figure 1A. *K. pneumoniae* was added to one liter of Luria–Bertani liquid medium and then incubated at 37 °C while shaking at 200 rpm until the culture’s OD600 value was close to 1.2. The bacterial cultures were subsequently subjected to centrifugation at 10,000× *g* for 30 min at 4 °C to eliminate precipitated cells. The resultant supernatant was concentrated using ultrafiltration in a tube with a 50 kDa retention capacity after being filtered using a 0.22 μm membrane (Millipore Corporation, Bedford, MA, USA). Following a 1:1 volume ratio of the concentrated solution to a 16% PEG 10,000 solution, the mixture was kept at 4 °C until the OMVs precipitated at the tube’s bottom. Finally, 1 mL of phosphate-buffered saline (PBS) was utilized to collect the precipitate.

This study used a washing process to improve the purity of rough *K. pneumoniae*_OMVs by washing them with 1 mL of PBS and centrifuging them at 15,000× *g* for 30 min at 4 °C. This process was repeated three times to effectively eliminate free lipopolysaccharide (LPS), heteroproteins, and other contaminants. Furthermore, to enhance the purification of *K. pneumoniae*_OMVs, the samples were diluted extensively with PBS and subsequently concentrated using a 10 kDa ultrafiltration device (Beyotime, Shanghai, China) prior to collection. This process was repeated three times to effectively eliminate residual PEG from the samples. The purified samples were thereafter kept at −80 °C for use in downstream investigations.

### 2.3. Characterization and Analysis of K. pneumoniae_OMVs

A total of 20 μL of the sample solution was pipetted onto a copper mesh slide in order to examine the ultrastructural properties of *K. pneumoniae*_OMVs. Subsequently, a 10 min incubation period was conducted at room temperature, after which the mesh was subjected to a 30 s rinse with distilled water. After removing excess liquid, a 2% uranium diacetate dihydrate solution was added drop by drop, followed by 20 μL of hydrogen peroxide in acetic acid solution for a one-minute negative staining treatment. Before being examined by transmission electron microscopy, the *K. pneumoniae*_OMVs were dried under an incandescent lamp for two minutes, and any remaining liquid was removed using filter paper. The existing *K. pneumoniae*_OMVs samples were diluted 50-fold for particle size analysis, which was performed using a nanoparticle detector. A Bradford assay (Bio-Rad Laboratories, Hercules, CA, USA) was used to measure the protein concentration. Additionally, 10% sodium dodecyl sulfate–polyacrylamide gel electrophoresis (SDS-PAGE) was used to separate proteins and assess their composition (Servicebio, Wuhan, China).

### 2.4. Mouse Immunisation and Infection

Eight-week-old *BALB/c* mice kept at the Yanbian Usniversity Laboratory Animal Center were used in this investigation. The Yanbian University Ethics Committee approved the experimental protocol, with approval number YD20240122001.

Mice immunized with *K. pneumoniae*_OMVs: Three groups of 18 *BALB/c* mice were chosen at random: normal control group (NC), 80 μg *K. pneumoniae*_OMVs-immunized group (80 μg OMVs), and 160 μg *K. pneumoniae*_OMVs-immunized group (160 μg OMVs). Each of the immunized groups received an intraperitoneal injection of 0.2 mL of *K. pneumoniae*_OMVs at doses of 400 μg/mL and 800 μg/mL, respectively, on days 7 and 21 after the initial immunization, whereas the NC group received an intraperitoneal injection of 0.2 mL of PBS. Blood samples were taken from each mouse’s tail vein on day 28 after the first immunization, and serum was then separated. The mice were then put to death by CO_2_ inhalation, and their hearts, livers, spleens, lungs, and kidneys were taken out for additional examination.

*K. pneumoniae* of sublethal-dose-infected animals: A total of 80 *BALB/c* mice were randomly assigned to four groups: normal control group (NC), *K. pneumoniae*-infected group (PBS), 80 μg *K. pneumoniae*_OMVs-immunoinfected group (80 μg OMVs), and 160 μg *K. pneumoniae*_OMVs-immunoinfected group (160 μg OMVs). Immunization: The groups receiving 80 μg and 160 μg of OMVs were immunized following the previously described protocol. In contrast, the NC group and the PBS group were administered intraperitoneal injections of an equivalent volume of PBS at the initial immunization, as well as on days 7 and 21, to serve as immunization controls. Infection: Except for the NC group, which received 0.2 mL PBS, all other groups were administered 0.2 mL of *K. pneumoniae* (4.2 × 10^5^ colony-forming units (CFU)) on the 29th day following the initial immunization. *P. mirabilis* (7.2 × 10^5^ CFU) was injected into 40 *BALB/c* mice that were immunized using a previously reported methodology in order to examine the specificity of the immunological protection offered by *K. pneumoniae*_OMVs. For seven days following infection, the daily survival of the mice in each group was monitored and documented.

### 2.5. Organ Coefficient and Bacterial Load

Following *K. pneumoniae*_OMVs immunization, the body weights of the mice in each experimental group were measured at 0, 7, 14, 21, and 28 days. Additionally, 28 days after immunization, the weights of the inoculated mice’s various organs were recorded. A particular formula (organ coefficient = organ weight (g)/mouse body weight (g) × 100%) was used to calculate the organ index. To look into the amount of germs in the livers and lungs of immunized mice 24 and 72 h after *K. pneumoniae* infection, 24 BALB/c mice were selected and randomly assigned to four groups, each receiving immunization in accordance with the previously outlined protocol. Organ harvesting was conducted in each group 24 and 72 h post-infection with *K. pneumoniae*, adhering to a standardized protocol. Subsequently, 0.1 g of tissue homogenate was prepared and inoculated into Luria–Bertani medium through serial dilution, followed by incubation at 37 °C for 12 h. The colony counting approach was used to quantify the bacterial burden.

### 2.6. Hematoxylin and Eosin Staining (HE) and Masson

After tissue samples were gathered, they were fixed for 48 h by submerging them in formalin solution. Following fixation, a series of graded alcohol solutions were used to dehydrate the samples, and xylene was then used to clear them. The specimens were then embedded in paraffin and sectioned into 4 μm slices. Staining was performed following the protocols outlined in the HE and Masson Staining Kit (Solebo, Beijing, China), and the generated sections were examined at 400× magnification using a light microscope.

### 2.7. RNA Isolation and Library Preparation and RNA Sequencing

Library Preparation and Sequencing: Twenty-eight days following the initial immunization, spleens from euthanized control mice and those administered 160 μg of *K. pneumoniae*_OMVs were harvested and promptly flash-frozen in liquid nitrogen. Total RNA was extracted from these tissue samples utilizing a Tritol RNA extraction kit (Beyotime, Shanghai, China). RNA integrity was evaluated using a 1% agarose gel electrophoresis assay using Biowest Agarose (Biowest, Nuaillé, France), and subsequent nucleic acid purification was carried out using an RNA Purification Kit (Shanghai Meiji Biomedical Technology, Shanghai, China). Library construction was performed using Illumina Stranded mRNA Prep Ligation reagent (Illumina, San Diego, CA, USA), adhering to stringent quality control protocols. RNA quality was further evaluated with an Agilent 2100 Bioanalyzer to ensure accurate assessment of RNA integrity. Sequencing was carried out using a NovaSeq Reagent Kit NovaSeqX Plus (Illumina). Post-sequencing, libraries were diluted to a concentration of 1.5 ng/μL as determined by a Qubit 2.0 fluorometer for initial quantification. The effective library concentration was precisely quantified via quantitative real-time PCR (qRT-PCR), confirming concentrations exceeded 1.5 nM to guarantee library quality. Metrics like O20, Q30, and GC content were computed for the clean reads after raw sequencing data was subjected to quality control using the fastp software (https://github.com/OpenGene/fastp (accessed on 28 May 2025)). Clean, high-quality filtered data was then acquired to guarantee the accuracy and dependability of following studies.

RNA-seq data analysis: The Norwood Biomedical Reference Transcriptome Cloud Analysis platform (https://magic.novogene.com/customer/main#/homeNew (accessed on 29 May 2025)) was used to analyze differentially expressed genes (DEGs). DEGs were identified based on the criteria of |log2 (fold change)| > 1 and a significance threshold of *p* < 0.05. Venn diagrams were generated to visualize overlapping DEGs, utilizing transcripts per kilobase per million mapped reads (TPM) as the expression metric. Additionally, volcano plots illustrating the distribution of DEGs were produced via the cloud analysis platform. Gene Ontology (GO) enrichment and Kyoto Encyclopedia of Genes and Genomes (KEGG) pathway analyses were used in subsequent functional annotation in order to clarify the predominant biological activities and signaling pathways relating to the DEGs, using a significance restriction of *p* < 0.05. All related datasets have been deposited in the NCBI database under accession number PRJNA1289660.

### 2.8. Assessment of Cell Viability and Cell Grouping

RAW264.7 cells were inoculated in 96-well plates at a density of 5 × 10^3^ cells/well and were cultured at 37 °C for 12 h under 5% CO_2_. Cell viability of RAW264.7 cells was subsequently assessed using a Cell Counting Kit-8 (CCK8, Beyotime, Shanghai, China) after treating the cells with DMEM containing 0.1, 1, 10, 20, 40, 80, and 100 μg/mL *K. pneumoniae*_OMVs for 24 h, respectively. In addition, RAW264.7 cells were inoculated into 6-well plates at a density of 2 × 10^6^ cells /well and were cultured in a 5% CO_2_ atmosphere at 37 °C for 12 h. Following this incubation, the original medium was replaced with a DMEM solution containing a specified concentration of PBS, along with 20 μg/mL and 40 μg/mL of *K. pneumoniae*_OMVs. The plates were then incubated for an additional 24 h at 37 °C. Ultimately, the cells and their supernatants were collected for further experimental analysis, with a minimum of three replicates established for each group.

### 2.9. qRT-PCR

To measure the mRNA expression levels of several cytokines and markers, including inducible nitric oxide synthase (iNOS), TNF-α, IL-6, CD86, IL-10, TGF-β, CD206, IFN-γ, T-bet, IL-4, GATA-3, and IL-2, cell and spleen tissue samples were taken from each experimental group. First-strand cDNA synthesis was carried out using gDNA Eraser reagent (Sangon Biotech, Shanghai, China) after total RNA was extracted using a total RNA extraction kit (Beyotime, China). Using synthetic cDNA as the template and GAPDH as the internal reference gene, qRT-PCR technology was used to measure the expression levels of these genes. In addition, the relative gene expression was calculated using the 2^-ΔΔCT^ method. The specific primers for each gene are detailed in the Supplementary Material, Table S1.

### 2.10. ELISA

Each group had serum or cell samples taken, and the ELISA method was used for analysis. Microtiter plate wells were coated with 40 μg of *K. pneumoniae*_OMVs and incubated at 4 °C for a whole night in order to detect particular IgG, IgG1, and IgG2a antibodies. Serum samples were diluted to 1:1500 in PBS as specified for use as the primary antibody and employed goat anti-mouse secondary antibodies specifically labelled with enzymes for IgG, IgG1, or IgG2a. Measurements were ultimately taken at OD450 in accordance with the reagent supplier’s specifications. TNF-α, IFN-γ, IL-4, IL-6, and IL-10, as well as the expression of T-bet, GATA-3, Arg-1, and NO, were measured using ELISA in accordance with the manufacturer’s instructions (MEIMIAN, Shanghai, China).

### 2.11. Western Blotting

SDS-PAGE was used to analyze protein extracts at concentrations of 6% and 12% under decreasing conditions. Following electrophoresis, the proteins were transferred to a PVDF membrane using a 200 mA current in a Tris–glycine fast buffer for an hour. After blocking the membrane for an hour with 5% BSA, diluted primary antibodies such as TNF-α, IFN-γ, IL-4, IL-6, IL-10, T-bet, GATA-3, Arg-1, iNOS, CD86, CD206, and IL-2 (1:1000, UpingBio, Hangzhou, China) were incubated overnight at 4 °C. Horseradish peroxidase (HRP)-conjugated goat anti-rabbit IgG (1:5000, Proteintech Group, Wuhan, China) was applied to the membrane after this incubation. An enhanced chemiluminescence (ECL) reaction was used to visualize the protein bands. The Image J 1.54f software was used to quantitatively analyze the bands using GAPDH as the internal reference protein.

### 2.12. IHC

IHC was used to detect the expression of TNF-α, IFN-γ, IL-4, IL-10, iNOS, and CD206 in spleen tissues. Paraffin sections of spleen tissue were soaked in xylene and graded alcohol and then incubated in sodium citrate buffer for 10 min. Moreover, the antigen repair was completed by heating to a boil using a microwave oven and then cooling to room temperature. Following 30 min of blocking with a 5% BSA solution, the sections were incubated overnight at 4 °C with a diluted primary antibody (1:100, UpingBio, Hangzhou, China). Following an hour of room-temperature treatment with a secondary antibody (1:5000, Proteintech Group, Wuhan, China), color development was accomplished using DAB chromogenic solution. Hematoxylin staining was performed, and the samples were examined under a light microscope at magnifications of 200× and 400×, with analysis conducted using the Image J (IHC) 1.54f software.

### 2.13. Statistical Analysis

GraphPad Prism 10 (GraphPad, San Diego, CA, USA) was used to analyze the data, and the results are shown as mean ± standard deviation (Mean ± SD). Either one-way or two-way analysis of variance (ANOVA) was used to evaluate group differences. The t-test was employed to evaluate differences between the two groups. Every experiment was carried out at least three times (*n* = 3), and a difference of less than 0.05 was considered statistically significant.

## 3. Results

### 3.1. Characterization of K. pneumoniae_OMVs

The *K. pneumoniae*_OMVs exhibited vesicular structures, as observed through transmission electron microscopy (Figure 1B). A nanoparticle size analyzer was used to determine the size distribution of *K. pneumoniae*_OMVs, which showed diameters ranging from 80 to 300 nm (Figure 1C). Furthermore, the average protein concentration of the isolated *K. pneumoniae*_OMVs was measured to be 650 μg/mL using the BCA assay. A total of 20 μg of the *K. pneumoniae*_OMVs sample was subjected to 12% SDS-PAGE electrophoresis, and three major bands of 40, 50, and 70 kDa were detected (Figure 1D).

### 3.2. Immunological Safety and Protective Effects of K. pneumoniae_OMVs

**Figure 2:**
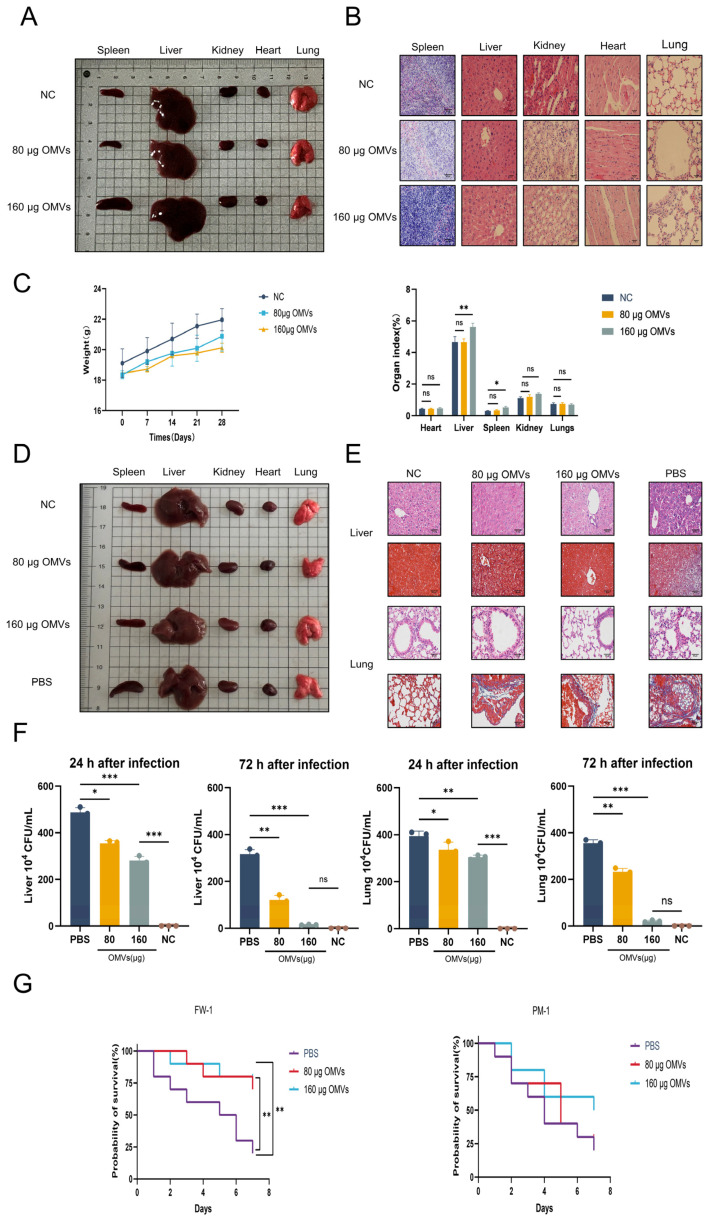
The immunological safety and protective effects of *K. pneumoniae*_OMVs. (**A**) Naked-eye observation of the spleen, liver, kidney, heart, and lung of immunized mice across different groups (*n* = 6). (**B**) The results of HE and Masson staining for the spleen, liver, kidneys, heart, and lungs of immunized mice in each group (400×, scale bar: 50 μm) (*n* = 6). (**C**) The effect of *K. pneumoniae*_OMVs on immunized mice’s body weight and organ coefficients (*n* = 6). (**D**) Naked-eye observation of the spleens, livers, kidneys, hearts, and lungs of *K. pneumoniae*-infected mice across the various groups (*n* = 6). (**E**) The livers and lungs of *K. pneumoniae*-infected mice in each group were stained with HE and Masson (400×, scale bar: 50 μm) (*n* = 6). (**F**) Bacterial burden in the lungs and livers of *K. pneumoniae*-infected mice at 24 and 72 h after infection in each of the three groups (*n* = 3). (**G**) Survival curves for mice infected with *K. pneumoniae* and *P. mirabilis* over a period of 7 days (*n* = 10). The mean ± standard error of the mean (SEM) is how the data are displayed. Either one-way or multifactor ANOVA was used to determine statistical significance, with significance levels denoted by * *p* < 0.05, ** *p* < 0.01, and *** *p* < 0.001, and ns refers to no difference.

In order to assess the immunological safety of *K. pneumoniae*_OMVs, a comprehensive examination of the primary organs of immunized mice was conducted, utilizing both visual inspection and microscopic analysis. The body weights and organ coefficients were measured and subjected to statistical evaluation. Visual and microscopic evaluations of the spleen, liver, kidneys, heart, and lungs in the 80 μg OMVs group revealed no significant alterations as compared to the NC group. Conversely, the 160 μg OMVs group exhibited notable splenic enlargement and a minor degree of inflammatory cell infiltration upon microscopic examination, which may be attributed to the pro-inflammatory response elicited by the LPS present in *K. pneumoniae*_OMVs (Figure 2A,B). Furthermore, *K. pneumoniae*_OMVs did not significantly impact the body weight or organ coefficients of the immunized mice in comparison to the NC group, except for an increase in liver and spleen organ coefficients observed in the 160 μg OMVs group (Figure 2C).

To investigate the immunoprotective effects of *K. pneumoniae*_OMVs against *K. pneumoniae* infection in mice, this study conducted macroscopic observations and histological analyses using HE and Masson staining on the primary organs affected by *K. pneumoniae* infection, namely, the liver and lungs. Naked-eye observation revealed significant splenic enlargement and renal congestion in the PBS group; in contrast, these conditions were markedly improved in the *K. pneumoniae*_OMVs-immunized group (Figure 2D). Histological results from HE and Masson staining indicated that, compared to the PBS group, the *K. pneumoniae*_OMVs-immunized group exhibited a notable reduction in inflammatory cell infiltration in the liver post-infection, with relatively intact hepatic sinusoids and lobular structures, and no significant tissue fibrosis was observed. Furthermore, the improvements in lung pathology were even more pronounced, as the *K. pneumoniae*_OMVs-immunized group displayed intact alveolar structures, thinner alveolar septa, reduced inflammatory cell infiltration, and decreased collagen fiber deposition, leading to a significant amelioration of fibrosis (Figure 2E).

Inhibition of bacterial proliferation represents an effective strategy employed by the host in response to infection. This study used colony-counting techniques to measure bacterial burdens in the livers and lungs of *K. pneumoniae*-infected mice in order to examine the bactericidal potential of *K. pneumoniae*_OMVs in eliciting immunological responses. The results indicated a significant reduction in bacterial loads in the livers and lungs of the *K. pneumoniae*_OMVs-immunized group 24 h post-infection compared to the PBS group (*p* < 0.01). Furthermore, at 72 h post-infection, the bacterial load in the group receiving 160 μg of OMVs approached zero, indicating near-complete bacterial clearance (Figure 2F). This study performed a statistical analysis of mortality rates among the different groups of mice within seven days post-*K. pneumoniae* infection since the immune protection rate is an essential reference for assessing immunoprotective efficacy. With a maximal protection rate of 80%, *K. pneumoniae*_OMVs dramatically improved the survival rate of inoculated mice infected with *K. pneumoniae*. The study used a sublethal dosage of *P. mirabilis* for infection in order to further clarify the specificity of the immune protection provided by *K. pneumoniae*_OMVs. The results showed that *K. pneumoniae*_OMVs provided limited protection, with a maximum efficacy of 50% (Figure 2G).

### 3.3. Analysis of Transcriptome Data

**Figure 3:**
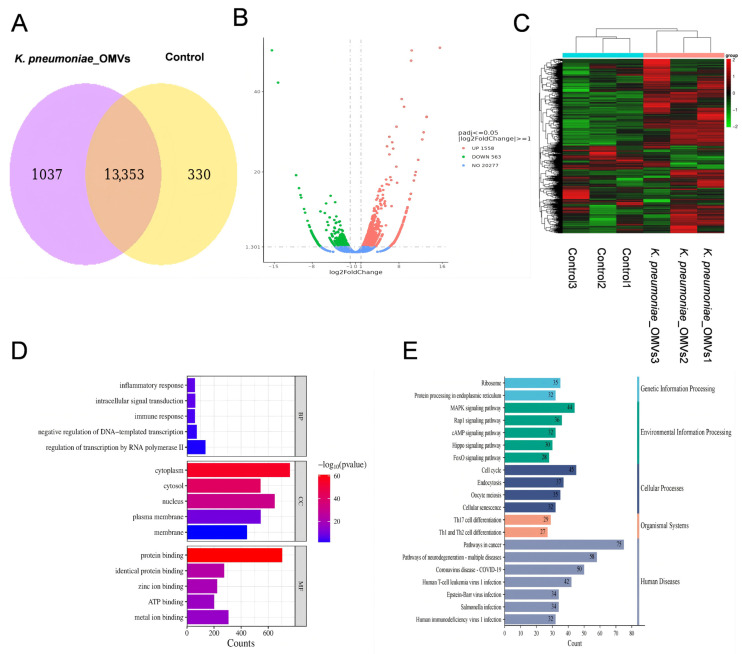
An analysis of transcriptome data derived from mice immunized with *K. pneumoniae*_OMVs. (**A**) A Venn diagram illustrating the co-expressed genes between the *K. pneumoniae*_OMVs and control groups. (**B**) A volcano plot depicting the DEGs. (**C**) A clustering heat map of the significant DEGs. (**D**) A bar graph representing the GO enrichment of the DEGs. (**E**) Summary plot of KEGG classification of DEGs. An online tool for data analysis and visualization, https://www.bioinformatics.com.cn (last viewed on 10 December 2024), created the heatmap [23].

A transcriptome analysis was performed to compare the group immunized with *K. pneumoniae*_OMVs (160 μg OMVs) with the control group in order to further understand the immunoprotective mechanisms induced by *K. pneumoniae*_OMVs. There were a total of 13,353 co-expressed genes in both groups (Figure 3A). There were 2121 DEGs in all, 1558 of which were upregulated and 653 of which were downregulated when comparing the *K. pneumoniae*_OMVs group to the control group (Figure 3B), and the significantly differentially enriched genes are shown in Figure 3C.

To better comprehend the molecular pathways behind immunity caused by *K. pneumoniae*_OMVs at the mRNA level, significantly differentially expressed genes were subjected to GO and KEGG enrichment analyses. Significant enrichment in biological processes (BP), cellular components (CC), and molecular functions (MF) was shown by GO analysis, as illustrated in Figure 3D. While the CC category was connected with cytoplasmic, cellular-nucleus-, and membrane-associated structures, the BP category was primarily involved in RNA transcriptional control, inflammatory response, and immunological response. The MF category included functions such as protein binding, zinc ion binding, and ATP binding. The KEGG analysis (Figure 3E) indicated that DEGs were significantly enriched in various critical disease and immune signaling pathways. The disease-associated pathways identified included the cancer pathway, the Salmonella infection pathway, and the MAPK pathway. In contrast, the Th17 cell differentiation pathway and the signaling pathways for Th1 and Th2 cell differentiation were significantly enriched in the immune-related pathways. These findings suggest that the immunoprotective mechanism elicited by *K. pneumoniae*_OMVs may operate through the activation of the innate immune system, involving macrophages, neutrophils, natural killer (NK) cells, and others, via the MAPK pathway, thereby influencing Th cells differentiation.

### 3.4. Effects of K. pneumoniae_OMVs on Raw264.7 Proliferation and Polarization

**Figure 4:**
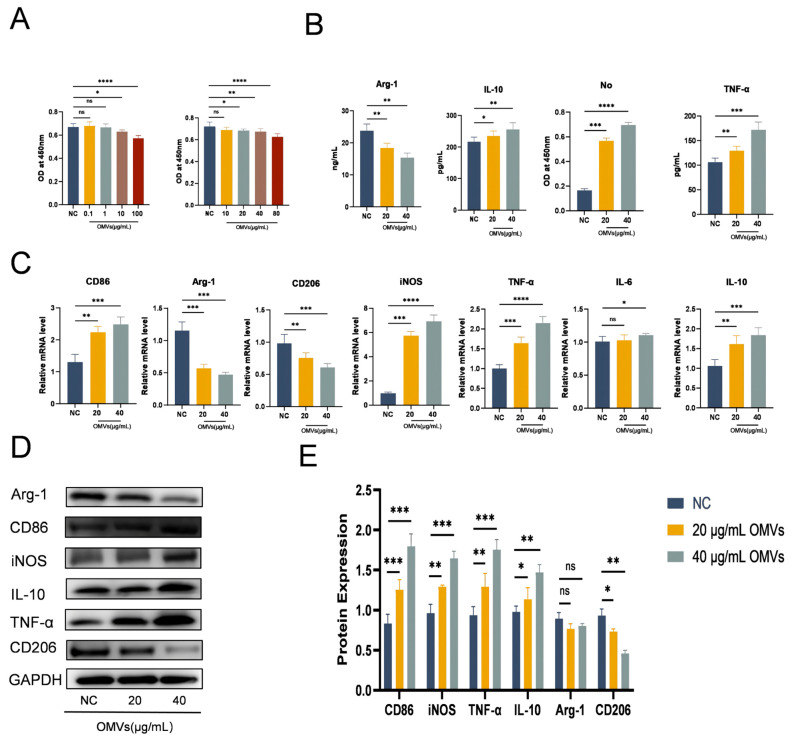
Effect of *K. pneumoniae*_OMVs on Raw264.7 proliferation and polarization. (**A**) Effects of 0, 0.1, 1, 10, 20, 40, 80, 100 μg/mL *K. pneumoniae*_OMVs on the viability of Raw264.7 macrophage cells (*n* = 3). Brown and red bar charts represent co-cultures of Raw264.7 macrophage cells with *K. pneumoniae* OMVs at different concentrations. (**B**) The influence of *K. pneumoniae*_OMVs on the secretion levels of cytokines, specifically Arg-1, IL-10, TNF-α, and NO, in Raw264.7 cells (*n* = 3). (**C**) The mRNA levels of CD86, Arg-1, CD206, iNOS, TNF-α, IL-10, and IL-6 in response to *K. pneumoniae*_OMVs (*n* = 3). (**D**,**E**) Arg-1, CD86, iNOS, IL-10, TNF-α, and CD206 protein expression levels in each of the three experimental groups. GAPDH is the internal reference protein. The mean ± SEM is how the data are displayed. Either one-way or multifactor ANOVA was used to determine statistical significance, with significance levels denoted by * *p* < 0.05, ** *p* < 0.01, *** *p* < 0.001, and **** *p* < 0.0001 and ns refers to no difference.

This study investigates the impact of *K. pneumoniae*_OMVs on macrophage polarization through co-culture experiments with macrophages. The impact of different *K. pneumoniae*_OMVs concentrations on Raw264.7 viability. After evaluating seven cells using the CCK-8 test, the experimental quantities of *K. pneumoniae*_OMVs were found to be 20 μg/mL and 40 μg/mL, as shown in Figure 4A. Following a 24 h treatment with the aforementioned concentrations of *K. pneumoniae*_OMVs, the ELISA results (Figure 4B) indicated a significant increase in the secretion levels of IL-10, TNF-α, and NO (*p* < 0.001). qRT-PCR analysis (Figure 4C) showed that the mRNA expression levels of CD86, TNF-α, IL-10, iNOS, and IL-6 were significantly higher in the 20 μg/mL and 40 μg/mL OMVs groups (*p* < 0.01) than in the NC group, although the levels of Arg-1 and CD206 were significantly lower (*p* < 0.01). These results were confirmed by Western blot analysis (Figure 4D,E), which revealed a significant decrease in the protein expression of Arg-1 and CD206 and a considerable rise in the protein expression of CD86, TNF-α, IL-10, and iNOS, in line with the qRT-PCR results. An analysis of the M1 polarization markers (iNOS and CD86), as well as the M2 polarization markers (Arg-1 and CD206), indicated that *K. pneumoniae*_OMVs can induce M1 polarization in Raw264.7 cells in a dose-dependent manner while suppressing M2 polarization. However, the significant elevation of IL-10 expression suggests the potential induction of M2b polarization by immune complexes.

### 3.5. K. pneumoniae_OMVs Induced Th Cells Differentiation in Mice

**Figure 5:**
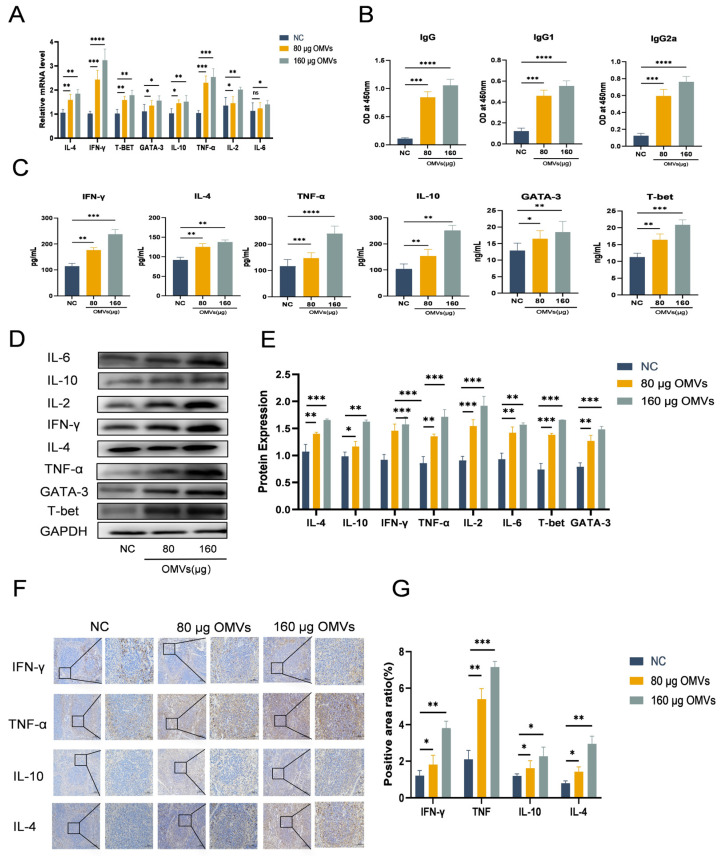
The impact of *K. pneumoniae*_OMVs on Th cell differentiation in murine models. (**A**) The impact of *K. pneumoniae*_OMVs on the mRNA expression levels of different transcription factors and cytokines, such as IL-4, IFN-γ, T-bet, GATA-3, IL-10, TNF-α, IL-2, and IL-6, in immunized mice (*n* = 6). (**B**) The impact of *K. pneumoniae*_OMVs on IgG, IgG1, and IgG2a secretion levels (*n* = 6). (**C**) The impact of *K. pneumoniae*_OMVs on IFN-γ, IL-4, TNF-α, IL-10, GATA-3, and T-bet secretion levels in immunized mice (*n* = 6). (**D**,**E**) Alterations in the protein expression levels of IL-6, IL-2, IL-4, IL-10, IFN-γ, TNF-α, T-bet, and GATA-3 across the different experimental groups. GAPDH is the internal reference protein (*n* = 6). (**F**,**G**) Immunohistochemical staining of IFN-γ, TNF-α, IL-10, and IL-4 in each group (200×, 400×, scale bar: 100 μm and 50 μm) (*n* = 6). The data are presented as the SEM ± mean. Statistical significance was determined using one-way or multifactor ANOVA. * *p* < 0.05, ** *p* < 0.01, *** *p* < 0.001, and **** *p* < 0.0001 and ns refers to no difference.

To examine the impact of *K. pneumoniae*_OMVs on the polarization of murine Th cells, this study established an in vivo immune model utilizing *K. pneumoniae*_OMVs. As shown in Figure 5A, the qRT-PCR results showed that both the 80 μg and 160 μg OMVs groups had significantly higher mRNA expression levels of IFN-γ, T-bet, IL-4, GATA-3, IL-2, IL-10, TNF-α, and IL-6 than the NC group. Additionally, as Figure 5B,C demonstrate, the ELISA results showed a significant rise (*p* < 0.01) in the levels of particular antibodies (IgG, IgG1, IgG2a) and cytokines (IFN-γ, IL-4, IL-10, TNF-α, T-bet, GATA-3).

IFN-γ, T-bet, IL-4, GATA-3, IL-2, IL-10, TNF-α, and IL-6 were among the proteins whose expression was significantly upregulated (*p* < 0.01) according to Western blot analysis (Figure 5D,E). Furthermore, immunohistochemical assessments (Figure 5F,G) indicated a notable increase in the expression levels of TNF-α, IFN-γ, IL-4, and IL-10, which corroborated the findings from the Western blot analysis. These findings imply that Th1 and Th2 cell types are successfully polarized by *K. pneumoniae*_OMVs. Additionally, the analysis of the IgG2a/IgG1 and IFN-γ/IL-4 ratios, which are indicative of shifts in Th1/Th2 balance, implies that *K. pneumoniae*_OMVs promote a dose-dependent shift towards Th1-type differentiation.

### 3.6. K. pneumoniae_OMVs Induce Macrophage Polarization and Helper T Cell Differentiation to Protect K. pneumoniae-Infected Mice

**Figure 6:**
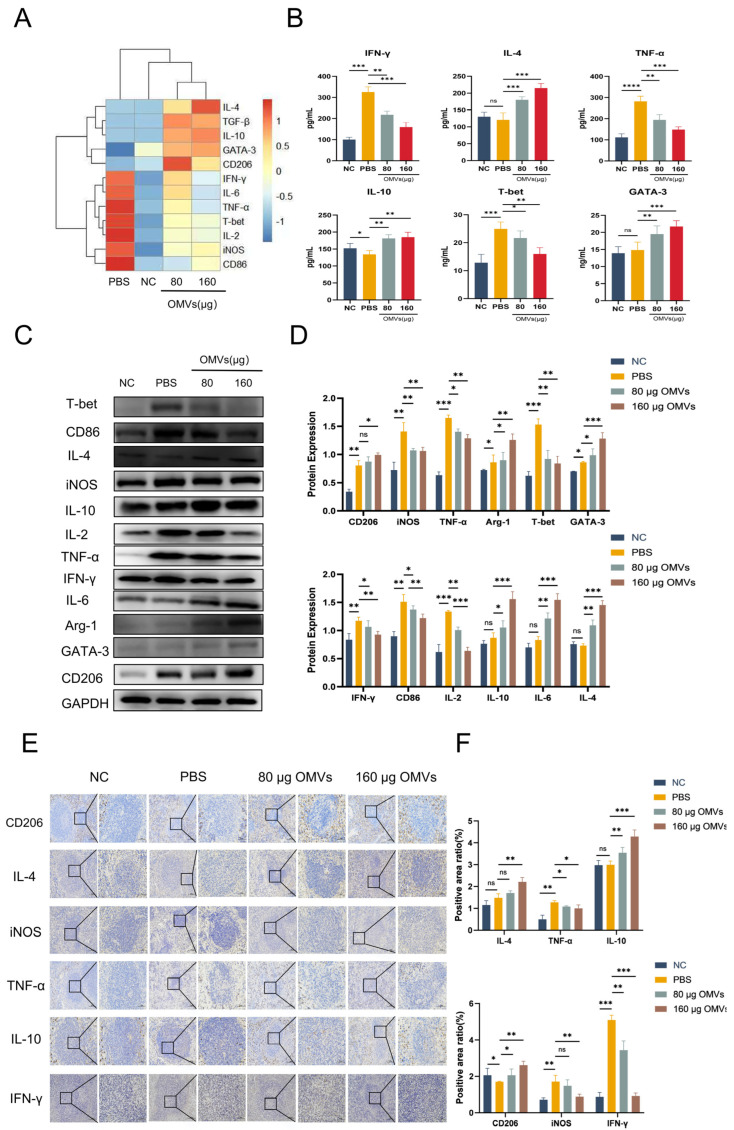
The polarization of macrophages and Th cells induced by *K. pneumoniae*_OMVs to confer protection in mice infected with *K. pneumoniae*. (**A**) The alterations in mRNA expression levels of IL-4, IFN-γ, T-bet, GATA-3, IL-10, TNF-α, IL-2, IL-6, TGF-β, CD86, CD206, iNOS, and Arg-1 across each group (*n* = 3). (**B**) The variations in the secretion levels of the cytokines IFN-γ, IL-10, T-bet, GATA-3, IL-4, and TNF-α in each group (*n* = 4). (**C**,**D**) Changes in the protein expression levels of IL-6, IL-2, IL-4, IL-10, IFN-γ, TNF-α, T-bet, GATA-3, CD86, CD206, iNOS, and Arg-1 were analyzed for each group. GAPDH is the internal reference protein (*n* = 3). (**E**,**F**) IHC staining of IFN-γ, TNF-α, IL-10, IL-4, iNOS, and CD206 in each group (200×, 400×, scale bar: 100 μm and 50 μm) (*n* = 3). The mean ± SEM is how the data are displayed. Either one-way or multifactor ANOVA was used to determine statistical significance, with significance levels denoted by * *p* < 0.05, ** *p* < 0.01, and *** *p* < 0.001, and **** *p* < 0.0001, and ns refers to no difference.

To investigate the changes in macrophage and Th cell polarization after *K. pneumoniae* infection in *K. pneumoniae*_OMVs-immunized mice, the *K. pneumoniae*_OMVs-immunized groups’ levels of IL-2, T-bet, TNF-α, IFN-γ, CD86, and iNOS mRNA were significantly lower than those of the PBS group, while the levels of IL-4, IL-10, IL-6, CD206, GATA-3, and TGF-β mRNA were significantly higher. Furthermore, the *K. pneumoniae*_OMVs immunoinfection groups’ secretion of pro-inflammatory cytokines TNF-α, IFN-γ, and T-bet was significantly lower (*p* < 0.001) according to the ELISA results (Figure 6B), while the secretion of inflammation-suppressive cytokines IL-4, IL-10, and GATA-3 was significantly higher (*p* < 0.001).

As shown in the results of Figure 6C,D, compared with the PBS group, the protein expression levels of the M1-/Th1-type polarization markers IL-2, T-bet, TNF-α, IFN-γ, CD86, and iNOS were significantly decreased in the 80 μg OMVs and 160 μg OMVs groups (*p* < 0.01); however, the Th2- and M2-type polarization markers IL-4, IL-10, IL-6, CD206, GATA-3, and TGF-β protein expression levels were significantly increased (*p* < 0.01). In addition, immunohistochemical results (Figure 6E,F) showed that the expression levels of CD206, IL-4, and IL-10 were significantly increased (*p* < 0.01), while the expression levels of TNF-α, IFN-γ, and iNOS were significantly decreased (*p* < 0.01), which was in agreement with the Western blotting results. In summary, the *K. pneumoniae*-infected group still showed M1/Th1 polarization after 7 days of infection, while the *K. pneumoniae*_OMVs-immunized group induced M2/Th2 polarization in a dose-dependent manner.

## 4. Discussion

In recent years, OMVs secreted by pathogenic bacteria have been considered a promising strategy to prevent bacterial infections. These vesicles carry complex and desirable antigenic molecules that modulate the host immune system, offering excellent protection against pathogenic infections [24,25]. However, OMVs contain high concentrations of LPS and other virulence proteins that can be cytotoxic [26]. Therefore, in addition to ensuring immunoprotection, immunosafety should be equally important to consider. We found that 200 μg and higher doses of *K. pneumoniae*_OMVs caused severe organ damage and even death in mice during immunization in the pre-experiment. Consequently, doses of 80 μg and 160 μg were selected for evaluation. While administration of the 160 μg dose led to splenomegaly and increased liver coefficients, indicative of pronounced systemic immune activation, it did not cause significant pathological tissue damage beyond the anticipated inflammatory cell infiltration. This highlights the necessity for precise dose regulation to elicit protective immunity while avoiding immunopathological harm, a fundamental consideration in the development of OMV-based vaccines.

Recently, there has been an alarming increase in the proportion of drug-resistant *K. pneumoniae* in various bacterial infections. It is capable of causing a wide range of pneumonias, liver abscesses, and urinary tract infections and lacks effective treatments [27,28]. Our study demonstrates that immunization with *K. pneumoniae*_OMVs substantially increases survival rates in mice, reaching up to 80%, following a lethal challenge with the homologous *K. pneumoniae* strain. This enhanced survival is corroborated by significant reductions in organ swelling and notable improvements in histopathological outcomes. However, the protective effect conferred by *K. pneumoniae*_OMVs is considerably diminished when challenged with heterologous strains, such as *P. mirabilis*. These findings are consistent with the recognized function of OMVs in presenting strain-specific antigens [29], indicating that while *K. pneumoniae*_OMVs offer robust protection against homologous strains, future vaccine development may necessitate engineered or combinatorial approaches to achieve broader cross-strain immunity [30].

Owing to the complex composition of OMVs, the precise mechanisms by which they elicit immunoprotection remain incompletely understood [31]. In the present study, transcriptomic analysis revealed that differentially expressed genes were enriched in immune-related pathways, notably the MAPK pathway and the pathways governing Th1 and Th2 cells differentiation. These pathways are critically involved in the polarization of macrophages and Th cells [32]. Prior research has established that *K. pneumoniae*_OMVs can be internalized by macrophages [33,34]. However, the specific macrophage phenotype induced has not been definitively characterized. Our in vitro experiments demonstrated that *K. pneumoniae*_OMVs induced a classical pro-inflammatory phenotype, evidenced by increased expression of TNF-α, IL-1β, and iNOS, indicative of M1-like polarization. Concurrently, within the *K. pneumoniae*_OMVs group, we observed a marked increase in both mRNA and protein expression levels of the anti-inflammatory cytokine IL-10, indicative of a potential M2b-like macrophage polarization. This observation stands in contrast to the effects elicited by certain other OMVs, such as those derived from *Escherichia coli* and *Porphyromonas gingivalis*, which predominantly provoke strong pro-inflammatory responses [35,36]. The existing literature suggests that factors including the specific molecular composition of OMVs—such as distinct lipoprotein or nucleic acid constituents—as well as the administered dose and duration of exposure, collectively influence the resultant macrophage phenotype. For example, engineered OMVs have been utilized to reprogram tumor-associated macrophages, underscoring their sophisticated capacity to modulate the immune microenvironment. Therefore, the ability of *K. pneumoniae*_OMVs to concurrently induce both pro-inflammatory and selective anti-inflammatory signals may be attributed to their unique compositional characteristics, facilitating the initiation of a more complex and finely regulated immune response [37,38]. This dual activation of signaling pathways observed in vitro was corroborated by in vivo findings, wherein *K. pneumoniae*_OMVs elicited a Th1/Th2 immune response with a bias toward Th1, as reflected by elevated IgG2a/IgG1 and IFN-γ/IL-4 ratios. It is noteworthy that Th1/M1 immune responses are generally associated with effective bacterial clearance.

Following infection with *K. pneumoniae*, *K. pneumoniae*_OMVs elicit a biphasic protective immune response. In the initial phase (24–72 h post-infection), bacterial burdens in the lungs and livers of mice immunized with *K. pneumoniae*_OMVs decreased rapidly in a dose-dependent manner. This effective bacterial clearance is associated with the prompt activation of an M1/Th1 immune response induced by *K. pneumoniae*_OMVs, which is essential for controlling acute *K. pneumoniae* infection [39,40]. Importantly, during the later phase (7 days post-infection), there was a significant upregulation of M2-/Th2-related markers, including Arg-1, IL-4, and IL-10, in *K. pneumoniae*_OMVs-immunized mice relative to the PBS group. This shift from a bactericidal M1/Th1 phenotype to a reparative M2/Th2 immune profile likely underlies the improved histopathological outcomes and survival rates observed, as it may reduce the sustained inflammatory damage commonly seen with antibiotic monotherapy [41,42,43]. Collectively, these findings indicate that the immune response modulated by *K. pneumoniae*_OMVs not only facilitates pathogen clearance but also promotes tissue repair, highlighting a significant advantage over treatments that solely exert bactericidal effects.

In conclusion, this study demonstrates that *K. pneumoniae*_OMVs administered at safe dosages can elicit a strong and specific protective immune response against lethal Klebsiella pneumoniae infections. The underlying protective mechanism is characterized by the initiation of a dynamic immune response, beginning with an M1-/Th1-dominant phase that facilitates rapid pathogen clearance, followed by a timely transition to an M2/Th2 phenotype that aids in resolving inflammation and promoting tissue repair. This orchestrated immune modulation underscores the advantages of OMV-based immunotherapy compared to traditional antibiotic treatments. The findings presented herein establish a solid theoretical basis for the development of *K. pneumoniae*_OMVs as viable vaccine candidates. Future investigations should aim to delineate the precise molecular mechanisms through which *K. pneumoniae*_OMVs influence macrophage and Th cell polarization while also striving to optimize these vesicles to enhance cross-protective efficacy against diverse strains.

## 5. Conclusions

In summary, this study provides evidence from both in vitro and in vivo experiments that *K. pneumoniae*_OMVs confer effective and sustained protection against *K. pneumoniae* infection in murine models. This protective effect is mediated through the promotion of adaptive polarization of Th cells and macrophages, resulting in an increase in survival rates up to 80%. Nonetheless, the protective efficacy of these OMVs against infections caused by heterologous strains is comparatively limited. These results offer a theoretical foundation for the development of vaccines based on *K. pneumoniae*_OMVs.

## Data Availability

The original contributions presented in this study are included in the article/Supplementary Material. Further inquiries can be directed to the corresponding author.

## Supplementary Materials

The following supporting information can be downloaded at: https://www.mdpi.com/article/10.3390/microorganisms13122849/s1. Table S1. Table qpcr primer.xlsx.
